# Dr. Evan Gabriel Evans

**Published:** 1916-03

**Authors:** 


**Dr. Evan Gabriel Evans,** Buffalo 1864, died at his home in Remsen, N. Y., Feb. 1, of asthma, aged 75. He was coroner of Oneida County for six years and a member of the board of supervisors for four years. He was also a clergyman.

